# Is it me? Self-recognition bias across sensory modalities and its relationship to autistic traits

**DOI:** 10.1186/s13229-015-0016-1

**Published:** 2015-03-30

**Authors:** Anya Chakraborty, Bhismadev Chakrabarti

**Affiliations:** Centre for Integrative Neuroscience and Neurodynamics, School of Psychology and Clinical Language Sciences, University of Reading, Reading, RG6 6AL UK

**Keywords:** Self-recognition, Autism, Self-face, Self-voice

## Abstract

**Background:**

Atypical self-processing is an emerging theme in autism research, suggested by lower self-reference effect in memory, and atypical neural responses to visual self-representations. Most research on physical self-processing in autism uses visual stimuli. However, the self is a multimodal construct, and therefore, it is essential to test self-recognition in other sensory modalities as well. Self-recognition in the auditory modality remains relatively unexplored and has not been tested in relation to autism and related traits. This study investigates self-recognition in auditory and visual domain in the general population and tests if it is associated with autistic traits.

**Methods:**

Thirty-nine neurotypical adults participated in a two-part study. In the first session, individual participant’s voice was recorded and face was photographed and morphed respectively with voices and faces from unfamiliar identities. In the second session, participants performed a ‘self-identification’ task, classifying each morph as ‘self’ voice (or face) or an ‘other’ voice (or face). All participants also completed the Autism Spectrum Quotient (AQ). For each sensory modality, slope of the self-recognition curve was used as individual self-recognition metric. These two self-recognition metrics were tested for association between each other, and with autistic traits.

**Results:**

Fifty percent ‘self’ response was reached for a higher percentage of self in the auditory domain compared to the visual domain (*t* = 3.142; *P* < 0.01). No significant correlation was noted between self-recognition bias across sensory modalities (*τ* = −0.165, *P* = 0.204). Higher recognition bias for self-voice was observed in individuals higher in autistic traits (*τ*_AQ_ = 0.301, *P* = 0.008). No such correlation was observed between recognition bias for self-face and autistic traits (*τ*_AQ_ = −0.020, *P* = 0.438).

**Conclusions:**

Our data shows that recognition bias for physical self-representation is not related across sensory modalities. Further, individuals with higher autistic traits were better able to discriminate self from other voices, but this relation was not observed with self-face. A narrow self-other overlap in the auditory domain seen in individuals with high autistic traits could arise due to enhanced perceptual processing of auditory stimuli often observed in individuals with autism.

## Background

The concept of ‘self’ has challenged thinkers and empiricists across disciplines, cultures, and time. A leading theoretical account of self-representation was proposed by William James [1]. According to this account, one of the key components of self is ‘material self’, the innermost part of which is the ownership of one’s own body. The ease of awareness of ‘bodily self’ or ‘physical self’ is fundamental to human social behaviour, since it enables the most basic distinction of self from other. This physical self-awareness emerges early and can be tested using mirror self-recognition in 18- to 24-month period in human infants [2]. Physical self-recognition has been suggested to be a precursor to the development of general self-awareness [3-5]. Self-awareness in turn is believed to share common underlying processes with mental state attribution and recognition of emotional state in others - aspects of behaviour allow for introspection, leading to the development of mentalizing/theory of mind (ToM) ability [6-9]. Consequently, the investigation of physical self-representation and recognition is fundamental to understanding the architecture of social behaviour, most forms of which require a distinction between self and other.

Physical self-representation is multimodal in nature and manifests across different senses and domains. Self-face, self-voice, self-body, and self-agency can all be regarded as instances of physical self-representation. However, most studies investigating self-other processing in the physical domain have used self-face processing as a metric of self-representation [10-12]. Understandably, this focus on visual representation of the physical self is based on the universal human ability to recognize ‘self-face’ from mirror-reflection and photographs. The investigation of the physical self as a multimodal construct, however, has been extremely limited. One study found that a combined presentation of self-face and self-voice inhibits (rather than facilitates) self-recognition, leading to the interpretation that visual self-face recognition is superior to auditory self-recognition [13]. In an fMRI study, Kaplan and others have shown overlapping patterns of activation in the inferior frontal gyrus during processing of both self-voice as well as self-face, suggesting a possible common neural correlate of multimodal physical self-representation [14]. However, there has been no direct behavioural test of physical self-representation across sensory modalities.

To address this gap in the literature, the first aim of our study was to systematically test multiple aspects of physical self-representation by measuring individual bias to both self-face and self-voice recognition. This line of enquiry tested how metrics of self-face and self-voice recognition compared across and within individuals. Self-face recognition has previously been tested by presenting self and other faces in a random order [7,10,15] as well as presenting morphed self and other faces [16]. We implemented a similar paradigm using self-other morphs in both visual (face) and auditory (voice) domains. The self-face and self-voice stimuli were morphed with unfamiliar faces and unfamiliar voices respectively to create domain-specific morph continua. Consequently, individual differences in bias for self-face (visual domain) and bias for self-voice (auditory domain) recognition were measured. The slope of the self-response curve was noted as morph levels shifted from self to other: a steeper curve indicated a narrower self-other categorization boundary. A narrower boundary theoretically corresponds to a reduced self-other overlap in the physical domain. The extent of this self-other overlap in the two modalities were correlated with one another to test whether physical self-representation is positively correlated across different sensory modalities. A reduced self-other overlap in the context of this experimental design theoretically corresponds to a more distinct physical self-representation. However, how this overlap varies across senses within individuals remains unexplored.

The second aim of our study was to explore individual differences in self-face and self-voice recognition and their association with autistic traits. Autistic traits are distributed continuously across the population, and individuals with autism spectrum conditions (ASC) score highly on these measures [17]. Importantly, trait measures of ASC have found to have the same aetiology at the extreme ends, suggesting that autistic traits provide a robust dimensional measure of autism-related symptoms in the general population [18]. Individuals with ASC exhibit deficits in different aspects of self-processing [19,20]. This has led to the proposal of an ‘absent self’ in autism, based on studies that show reduced memory for self-relevant words [21,22], reduced self-other discrimination in the ventromedial prefrontal cortex [19], and diminished autobiographical memory in autism [23,24]. However, none of these previous reports have directly tested psychophysical metrics of physical self-representation in relation to autistic traits.

Accordingly, we investigated how these measures of physical self-representation were mapped onto traits related to autism. However, in the absence of any directly relevant prior evidence, we did not have a hypothesis about the directionality of this relationship.

## Methods

### Participants

Thirty-nine White Caucasian participants aged between 18 and 40 years were recruited in the study (10 males, 29 females, age = 23 ± 4.5 years). Only White Caucasian participants were chosen since the ‘other’ faces were constant across participants and were of this ethnicity. All participants had normal or corrected to normal vision and hearing and were right handed. Participants took part in a two-part experiment (face and voice), the order of which was counter-balanced across participants. Three participants did not complete the voice part of the experiment due to technical issues. Participants also completed the Autism Spectrum Quotient (AQ) questionnaire online (18.59 ± 7.55). All participants signed a consent form giving their consent to taking part in the study. The study was approved by School of Psychology and Clinical Language Sciences Ethics Committee, University of Reading.

### Self-face identification

#### Stimuli

Stimuli were individually tailored for each participant. Each participant was photographed using a digital camera (Toshiba Camileo S30, Toshiba Corporation, Tokyo, Japan) in identical conditions under artificial lighting. Four volunteers (2 males for male participants and 2 females for female participants) were selected to serve as ‘unfamiliar faces’ were also photographed under the same conditions. Participants looked directly at the camera and were seated at a distance of 100 cm with a white background while holding a neutral expression. The photographs were then converted to grayscale and external features (hairline, jawline, and ears) were removed. This photograph was then mounted on an oval frame and cropped to a dimension of 350 × 500 pixels using GIMP [25]. Two sets of stimuli were created for each participant face, by morphing with two ‘unfamiliar faces’ using Sqirlz Morph (Xiberpix, Solihull, UK) [26].

**Figure 1:**
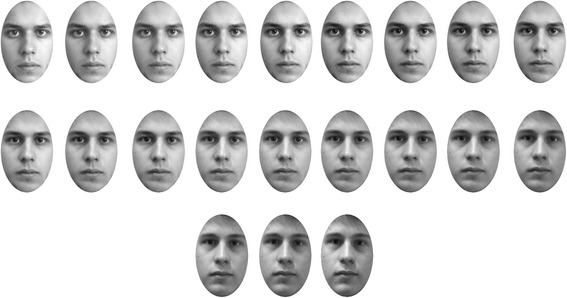
**Stimuli set representing face morphs at 5% step size.**

Each face morph continuum had 21 images at 5% step sizes (100% self, 95, 90……….10, 5, 0% self) (Figure 1). In the test phase, images were presented at a viewing distance of approximately 55 cm, on a 30.5 cm × 23 cm inch colour TFT active matrix XGA LCD monitor (1,024 × 768 pixels) run at 60 Hz by a PC.

#### Procedure

**Figure 2:**
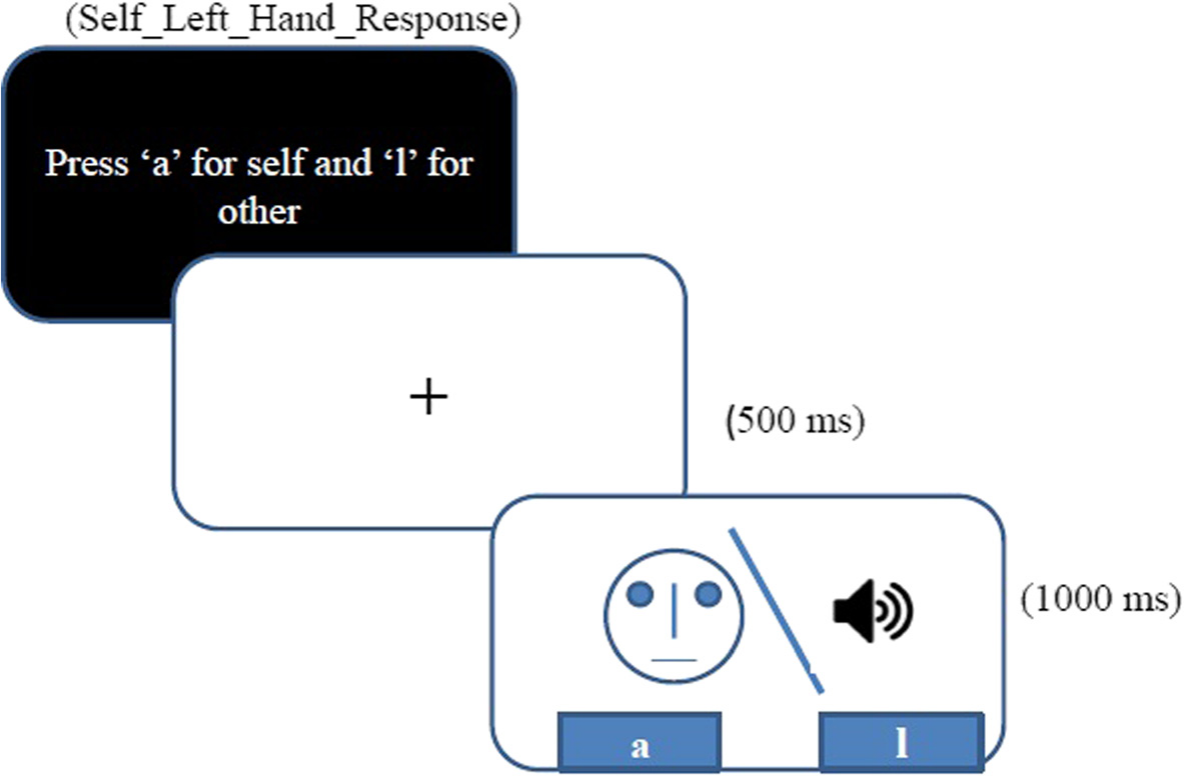
**Task design and an example trial from face/voice block.**

The test run comprised of 2 blocks, each consisting of 2 sets of ‘self-unfamiliar’ morph continuum of 44 images in total, presented twice in a randomized manner. Each block had a total of 88 trials, and thus, each run had a total of 176 trials. Each trial consisted of a cross-hair presented for 500 ms followed by the stimuli which lasted for 1,000 ms during which participant had to log in the key-press response. Participants had to classify each image as ‘self’ or ‘other’ by pressing ‘a’ key for self-face (left-hand self-response) and ‘l’ key for other face in one block, and the response keys were reversed in the next block (right-hand self-response) (Figure 2). This order was counter-balanced across participants. Participants were asked if the ‘unfamiliar face’ was truly unfamiliar, at the end of the experiment. None of the participants reported being familiar with either of the ‘unfamiliar’ faces.

### Self-voice identification

#### Stimuli

Stimuli were individually tailored for each participant. Each participant’s voice was recorded and digitized at 44.1 kHz in a sound-proof booth using a high-resolution microphone and Adobe Audition [27]. Each recording was made as participants uttered a train of monosyllable /ba/ in a neutral voice, at the rate of 1 syllable/s. This was chosen as the stimulus to avoid differences due to accents and semantic information that can influence self-voice recognition from sentences. Additionally, using syllabic trains avoids confounds due grammar, syntax, and psychological characteristics of other speakers that people focus on when hearing their own voice [28]. Two gender-matched unfamiliar/other voices were also recorded under similar conditions.

Each voice train was trimmed to one single /ba/ utterance of 1,000 ms, followed by noise removal, equalization (filter of 3 dB), and normalization to peak volume of 0 dB using Audacity [29]. The preprocessed voice stimulus was then morphed with the unfamiliar voice using STRAIGHT [30] signal processing package implemented in Matlab [31]. Two sets of morphing continua each of the 11 voice excerpts were thus created (from 0% to 100% in steps of 10%).

#### Procedure

The test run comprised of 2 blocks, each consisting of 2 sets of ‘self-unfamiliar’ voice morph continuum consisting of 22 stimuli in total, presented twice in a randomized manner. Each block had a total of 44 trials, thus making each run consists of 88 trials. Each trial consisted of a cross-hair presented for 500 ms followed by the stimuli which lasted for 1,000 ms during which participant had to log in the key-press response (Figure 2). Participants used a similar button press task to identify a voice as self/other (as in the face task). No participants reported being familiar of either of the two ‘unfamiliar’ voices.

The order of face and voice tasks was counter-balanced across participants. Both tasks were run using E-Prime version 2.0 [32]. Following the entire experiment, each participant had to rate the perceived visual/auditory similarity between the 100% self (face and voice) and the 50% self (face and voice) with the respective 2 ‘unfamiliar’ faces and voices. This was to ensure that perceived similarity to the unfamiliar faces or voices by the participant did not bias the ‘self-classification’ response. The 50% morph was chosen because morphing techniques can create morphs that may appear to look more similar to one face or another across individuals at the same morph level. This was done in order to test if there was a difference in similarity ratings across participants in explicit appraisal of 50% morph similarity to self or other.

### Data analysis

**Figure 3:**
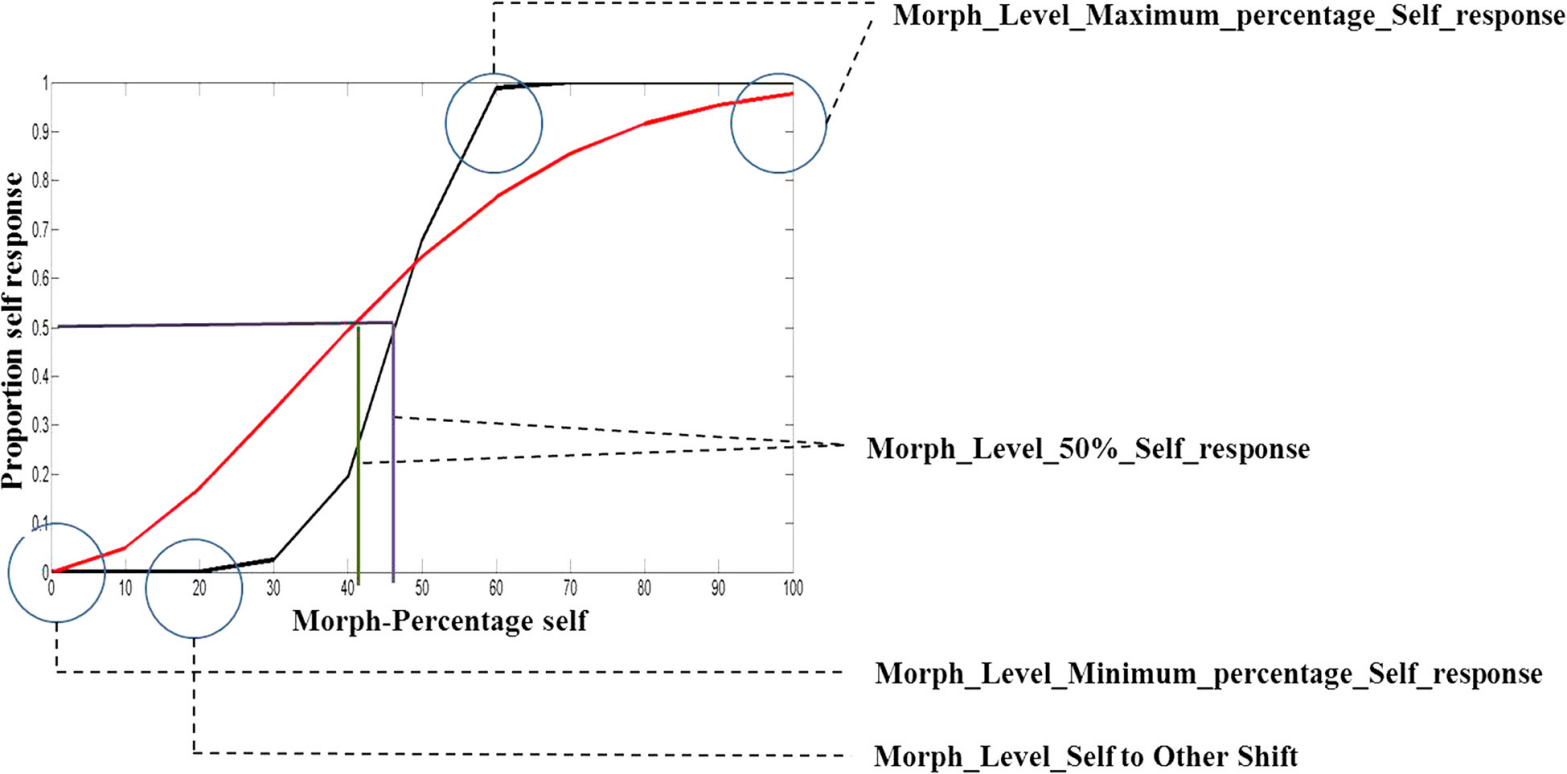
**Schematic representation of parameters characterized and analysed for the physical self-representation data.** The red curve represents a shallow slope in which the shift from ‘other’ to ‘self’ labelling occurs over a broader range of change in stimulus features. Correspondingly, the steeper curve in black represents a change label over a narrow range of change in stimulus features.

For each level of morph, the percentage self-response (that is, how often was a given morph labelled as ‘self’) was recorded, and a response curve was generated (separately for face and voice). For each modality, the percentage ‘self-response’ was normalized within participants, to account for baseline differences in self-recognition. The slope for each participant, for each modality, was calculated using a psychometric function fitted for maximum likelihood estimation for Weibull distribution. Depending on the task, the psychometric function gives a steep or shallow slope (Figure 3). The steepness of this slope is interpreted as an extent of overlap between the self-face/voice and other face/voice representation. A steeper slope indicates a reduced overlap between the self and other representation. In other words, a steeper slope represents a more distinct self-representation.

Statistical analyses were conducted using SPSS [33] and R software [34].

## Results

### Physical self-representation

#### Parameters

To characterize the distribution of self-response across two modalities, we calculated the morph level at which 50% self-response was recorded. A paired *t*-test revealed that the morph level at which self-face response reached 50% was lower (i.e. containing lower percentage of self) compared to the morph level at which 50% self-voice response was reached (*t* = −3.142, *P* = 0.003) (mean morph level for face = 40.57, sd = 11.87; mean morph level for voice = 46.81, sd = 0.248). The maximum percentage labelled as self was comparable across two modalities. Furthermore, the morph level at which the shift occurs from the label of ‘self’ to ‘other’ for both modalities across all participants was calculated (see Table 1).

**Table 1:** **Distribution of self-response (%) parameters for face and voice morphs and slopes of the corresponding psychometric functions**

**Modality (mean (SD); range)**	**Maximum self-response (%)**	**Minimum self-response (%)**	**Morph level for self to other shift (%)**	**Slope**
Face	97.05 (8.73);100–62.5	0	44.12 (12.09)	7.63 ± 0.36
Voice	87.87 (14.9); 100–62.5	18.94 (17.01); 50 - 0	27.12 (14.14)	7.23 ± 1.25

The self-other overlap was characterized using the slope of the psychometric function as described earlier. To test the overlap between self and other representation in the two sensory domains exhibited any relationship, Kendall rank correlation coefficient (two-tailed) was calculated between the slopes of self-face and self-voice recognition. The choice of the test was made as neither of the slope variables for faces and voices showed a normal distribution (Shapiro-Wilk test *P* < 0.001). This analysis included only the participants who completed both auditory and visual tasks. After analysis for outliers, there was no significant correlation between slope for self-face and self-voice recognition (*τ*(35) = −0.165, *P* = 0.204). A partial Kendall correlation coefficient was calculated controlling for gender (to account for the unequal male to female ratio). This analysis did not alter the results (face slope and voice slope: *τ* = −0.163, *P* = 0.175). To further elaborate on this null result, Bayes factor was computed. The Bayes factor for this correlation was 0.70, indicating barely any evidence for the hypothesis that physical self-representation across modalities are correlated [35].

#### Physical self-representation and autistic traits

To evaluate individual differences in physical self-representation across sensory domains and autistic symptoms, Kendall rank correlation coefficient was calculated between the following variable pairs:

- Visual self-other representation and autistic traits – (face slope and AQ scores)
- Auditory self-other representation and autistic traits – (voice slope and AQ scores)

**Figure 4:**
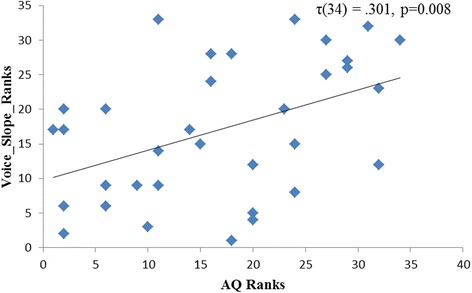
**Rank correlation between slope of self-response curves of voice and autistic traits (AQ).**

Outliers (defined as cook's d > 4/N where N is the number of participants) were removed prior to analysis. All p-values reported are one-tailed, in light of the directional nature of the hypotheses. Auditory self-representation was found to be positively correlated to the AQ scores (*τ*_AQ_(34) = 0.301, *P* = 0.008 (Figure 4). The Bayes factor value of 3.10 indicates substantial evidence for the theory that there is a correlation between auditory self-representation and autistic traits. Visual self-representation was not significantly correlated with AQ scores (*τ*_AQ_(36) = −0.020, *P* = 0.438). The corresponding Bayes factor value of 0.55 indicates barely any evidence for the theory that visual self-representation and autistic traits are related. The data was further analysed with gender as a covariate (accounting for the unequal male to female ratio). This analysis revealed a very similar pattern of results to those reported above (face slope and AQ: *τ* = −0.007; *P* = 0.95; voice slope and AQ: *τ* = 0.301; *P* = 0.015).

To check if the pattern of response was biased by specific ‘other’ faces/voices (since two ‘other’ faces/voices were used), *t* statistics were computed using a paired sample *t*-test for percentage self-response between two unfamiliar faces and two unfamiliar voices for each participant. This analysis revealed no significant differences at *P* < 0.05(faces: *t* = 0.349; *P* = 0.727; voices: *t* = 1.608; *P* = 0.109).

## Discussion

This study tested (a) if physical self-representation is comparable between visual and auditory modalities and (b) if autistic traits are associated with sensory modality-specific self-representation.

Physical self-representation was measured as the slope for self-recognition, varying as a function of available physical self-related information. Available physical self-related information was manipulated in both visual and auditory domains by creating degrees of morphs with differing percentages of self-related information. The steepness of the slope, calculated from the self-recognition responses across the different degrees of morphs, provided a measure of stimulus range over which the participant shifts between categories. A steeper slope indicates narrower range and a reduced overlap between self and other. This metric was then compared across the modalities and with the autistic traits.

We found that physical self-representation across the auditory and visual domains did not correlate with each other. In other words, individuals with a narrower self-other overlap in visual domain (or more distinct self-face representation) did not show a correspondingly narrow self-other overlap in the auditory domain. This observation suggests that physical self-representation is not unitary across sensory modalities. While common brain regions such as the inferior frontal gyrus IFG might be involved in responding to both self-face and self-voice [14], this result suggests that the bias to self-related signals in the different sensory modalities might be sufficiently distinct. However, these differences in self-recognition bias across sensory modalities do not invalidate the possibility that physical self-related information may be processed in an integrated multisensory manner [3,36].

Our results further show that individuals with high autistic traits show narrower self-other overlap in the auditory domain than in the visual domain. The steeper slope in the auditory domain for individuals high in autistic trait indicates that the stimulus features allowed such individuals to shift categories (from ‘self’ to ‘other’) over a narrow range. This suggests that a narrow representation of self-voice (or a more distinct representation of self-voice) is associated with higher autistic traits. One interpretation of this result is that individuals high in autistic traits have a ‘narrower’ physical self-representation. This narrow physical self-representation can be interpreted such that any deviation from it is perceived to be an ‘other’, making it difficult to simulate others. This is particularly interesting, since flexibility of self-representation can be useful in order to put oneself in another person’s shoes (that is, simulate them). However, this relationship of high autistic traits and narrower physical self-representation was seen only for self-voice stimuli, and not for self-face stimuli. One potential mechanism through which a more distinct physical self-representation can be instantiated is through heightened attention to interoceptive cues, as has been noted by a recent study in individuals with ASC [37].

Since the relationship of autistic traits and narrower physical self-representation is only seen for self-voice and not for self-face, an alternative explanation based on the sensory characteristics of self-face and self-voice stimuli is offered here. In contrast to faces, our familiarity with our own voices as it sounds to others is usually lower. This is because we hear our own voices through bone conduction, which sounds different from that we can hear from recorded self-voice that we hear through air conduction. Previous reports have suggested that individuals focus on the grammar, syntax, and psychological characteristics of other speakers, while they focus on the tonal qualities when hearing their own voice [28]. The nature of the voice stimuli in our experiment was also devoid of any semantic information, a feature that makes recognition of self-voice further pitch dependent. The tonal qualities are more pitch dependent, and higher abilities in pitch discrimination are reported in autism [38]. It is therefore possible that the higher perceptual functioning in the auditory domain, often seen in autism, may underlie the better recognition of self in the auditory domain by individuals with high autistic traits.

It should be noted that the current study sample was not balanced for gender and did not have sufficient power for the analyses to be stratified by gender. Notwithstanding this limitation, controlling for gender in a separate correlation analysis did not change the reported results. However, in view of a female advantage suggested in an early study based on polaroid photographs of self-faces [39], future work should further test the role of gender in self-face and extend it to self-voice recognition. Specifically for self-voice recognition, future experiments should test the competing explanations of the results presented in this study, by testing if the better discrimination of pitch in unrelated control sounds can account for this observed positive correlation of self-voice recognition bias and autistic traits.

Self-representation in the psychological domain has been investigated widely in recent behavioural and neuroimaging studies [40,41]. It will be of interest to test the relationship of physical self-representation with self-representation in the psychological domain. In addition, cultural differences in these different aspects of self-representation and how these are altered in psychopathological conditions such as ASC need to be addressed by future studies.

## Conclusions

In this study, we showed that recognition bias for physical self-representation across the visual and auditory domain is not a unitary or correlated phenomenon. We also showed that recognition bias for self-voice is correlated with autistic traits, such that individuals with high autistic traits show a narrow self-other overlap. Future experiments should include non-voice stimuli to test between competing interpretations suggested in this report and extend the paradigm to other cultures as well as individuals with autism spectrum conditions.

## Abbreviations

ASQ: Autism Spectrum Quotient
ASC: autism spectrum condition
fMRI: functional neuroimaging
IFG: inferior frontal gyrus
TOM: theory of mind
